# A Pilot Study into the Effects of the CB1 Cannabinoid Receptor Agonist WIN55,212-2 or the Antagonist/Inverse Agonist AM251 on Sleep in Rats

**DOI:** 10.1155/2011/178469

**Published:** 2012-01-04

**Authors:** Anushka V. Goonawardena, Andrea Plano, Lianne Robinson, Bettina Platt, Robert E. Hampson, Gernot Riedel

**Affiliations:** ^1^Department of Physiology and Pharmacology, Wake Forest University School of Medicine, Winston-Salem, NC 27157-1083, USA; ^2^School of Medical Sciences, University of Aberdeen, Aberdeen AB25 2ZD, UK; ^3^Institute of Medical Sciences, University of Aberdeen, Foresterhill, Aberdeen AB25 2ZD, UK

## Abstract

The plant cannabinoid Δ^9^-tetrahydrocannabinol and the endocannabinoid anandamide increase the amount of sleep via a CB1 receptor mediated mechanism. Here, we explored the use of a novel electroencephalogram (EEG) recording device based on wireless EEG microchip technology (Neurologger) in freely-moving rats, and its utility in experiments of cannabinoids-induced alterations of EEG/vigilance stages. EEG was recorded through epidural electrodes placed above pre-frontal and parietal cortex (overlaying the dorsal hippocampus). As cannabinoids, we acutely administered the full synthetic CB1 receptor agonist, WIN55,212-2 (1 mg/kg), and the antagonist/inverse agonist, AM251 (2 mg/kg), either alone or together through the intraperitoneal route. WIN55,212-2 increased the total amount of NREM sleep and the length of each NREM bout, but this was unlikely due to CB1 receptor activation since it was not prevented by AM251. However, WIN55,212-2 also lowered overall EEG spectral power especially in theta and alpha frequency bands during wakefulness and NREM sleep, and this effect was reversed by AM251. The antagonist/inverse agonist caused no sleep alterations by itself and moderately increased spectral power in Theta, alpha and beta frequency bands during NREM sleep when administered on its own. Implications of endocannabinoid modulation of the sleep-wake cycle and its possible interactions with other transmitter systems are considered.

## 1. Introduction

It is widely known that the active ingredient of marijuana, Δ^9^-Tetrahydrocannabinol (Δ^9^-THC), modulates the sleep-wake cycle. During the 1970s and 1980s, several experiments carried out in humans and rats demonstrated that Δ^9^-THC was able to increase sleep [1–5]. Similar effects can be evoked by the endocannabinoid arachidonylethanolamide (anandamide), including the modulation of food intake, body temperature, locomotor activity, pain perception, sexual behavior, learning and memory, and sleep [6, 7]. Santucci and coworkers [8] were the first to study the physiological role of endocannabinoids on sleep. They systemically administered the CB1 receptor antagonist/inverse agonist, SR141716A (SR), to rats and observed a dose-dependent increase in wakefulness (W) and a reduction in both slow-wave (SWS) and rapid eye movement (REM) sleep. This indicated that the wake-promoting properties of SR arise as a result of the inhibition of the endocannabinoid tone on CB1 receptors [9] and/or due to an inverse agonism [10, 11] on the same subset of G-protein coupled receptors. Indeed, endocannabinoids such as anandamide also increased non-REM (NREM) and REM sleep following acute intracerebroventricular (icv) administration in rats [12]. More specifically, it appears that sleep modulations occur through CB1 receptors in the peduncular pontine tegmental nucleus [13]. Elevation of anandamide by the putative inhibitor of its transporter, VDM-11 [14], and direct administration of endocannabinoids to these sleep modulating areas [13] induced increases in sleep, which were blocked by SR in rats, implicating CB1 receptors in this action.

To date, no study has examined how full CB1 receptor agonists modulate the sleep-wake cycle in rats. Hence, the main objective here was to pharmacologically characterize the acute effects of the full CB1 receptor agonist WIN 55,212-2 (WIN-2) on sleep. This is of particular interest since we have previously reported WIN-2 to impair memory formation in spatial learning tasks [15–17], and these deficits are reversible by CB1 receptor antagonist/inverse agonist such as AM251. We, therefore, reasoned that sleep anomalies evoked by WIN-2 if mediated by CB1 could be reversed by AM251.

An additional novelty of this study arises from the application of a novel wireless multichannel EEG data logging device which has been developed for use in mice. We here adapted the system to rat in a proof-of-principle pilot study and at the same time explored the possibility to reduce the number of subjects by repeatedly monitoring drug effects in a longitudinal fashion in freely moving rats.

## 2. Methods and Materials

### 2.1. Animals

Adult, male Lister-Hooded rats (Harlan, UK) aged 7-8 weeks and weighing 250–300 g were individually housed at a constant temperature (23 ± 1°C) and a 12:12-hour light-dark cycle (lights on from 07:00 to 19:00) with free access to food (rat chow) and water. All subjects were habituated in their home cages for at least 3 days prior to administration of drugs and EEG recordings. All experiments were performed under UK Home Office regulations and in accordance with the Federation of European Laboratory Animal Science Associations (FELASA) guidelines.

### 2.2. Surgery

Animals were anesthetized using isoflurane (induction 3%, maintenance 1.2–1.5%) and the protocol followed the one reported previously for mice [18]. Coordinates were adjusted according to the rat stereotaxic atlas [19] and epidural gold screw electrodes placed above the medial prefrontal cortex (3 mm anterior to Bregma, close to midline) and parietal cortex overlaying both left and right hippocampi, respectively (2.8 mm posterior to Bregma, ±3.3 mm lateral to midline).

All animals were allowed to recover for at least 7–10 days before experiments commenced during which analgesics were administered for the first 2 days.

### 2.3. Drug Groups

WIN55,212-2 and AM-251 (Tocris, Bristol, UK) were dissolved in vehicle [Triethylene glycol (TEG) and phosphate buffered saline (PBS); 50 : 50 vol/vol] and intraperitoneally administered at midday (12pm) followed by the start of EEG recording (total length: 6 hours). With one week washout in between, all animals (*n* = 4) were administered with vehicle, WIN55, 212-2 (1.0 mg/kg), AM-251 (2.0 mg/kg), and WIN-2 (1.0 mg/kg) + AM251 (2.0 mg/kg) (i.e., within-subject study; same drug(s) for all rats; intraperitoneal injection; WIN-2 and AM251 coadministered; total volume 5 mL/kg). We were particularly interested in drug doses which in our previous behavioural studies have shown efficacy and impaired memory formation [15–17]. A full-dose response relationship was beyond the scope of this pilot study. 

### 2.4. EEG Recording and Analyses

A wireless data logger (Neurologger; New Behavior, Zurich, Switzerland) was used to register the EEG activity from freely behaving rats. It was connected to a 7-pin head-stage and recorded three channels at a sampling rate of 200 Hz with filters set to 0.1 Hz (high-pass) and 70 Hz (low-pass). A built-in accelerometer monitored movements. Data were downloaded offline to a PC using USB plug-in docking stations [18].

Data retrieved were transformed with Matlab 7 (The MathWorks Inc., Natick, USA) and imported into SleepSign (Kissei Comtec Co. Ltd, Nagano, Japan) for vigilant staging and extrapolation of spectral power as previously defined [18]. Vigilance stages (Wakefulness, NREM, and REM sleep) of 4 sec epochs were identified by Fast Fourier Transform (FFT; delta/theta ratio from parietal EEGs). Accelerometer activity (body movement) and automated staging (based on accelerometer activation and frequency dominance; delta for NREM, theta for REM sleep) was followed by visual inspection and corrections to exclude any movement-related artifacts. The EEG power spectra were calculated based on FFT of each 4 sec epoch for each vigilance stage, normalized relative to the absolute maximum power over all frequency bands (1–20 Hz) and averaged for each drug group for hippocampus and prefrontal cortex (spectral bands: delta: 0.5–5 Hz, theta: 5–9 Hz, alpha: 9–14 Hz and beta 14–20 Hz). Sleep scoring and all power spectral analyses were carried out by a single examiner unaware of the rat's treatment group. 

### 2.5. Statistical Analyses

Statistical significance of all vigilance state parameters (i.e., total time; average length awake, NREM, and REM events; latencies to 1st NREM and 1st REM episodes) was assessed using repeated measures one-way analysis of variance (ANOVA) followed by Bonferroni post hoc multiple comparisons between different treatment groups using GraphPad Prism version 5.0 (GraphPad Software Inc., San Diego, CA, USA). For EEG power spectral analyses, a two-way factorial ANOVA was conducted using treatment (drug) group and frequencies as discriminators. Post hoc planned paired comparisons on selected frequency bands were carried out to determine sources of overall significance. All data are expressed as mean ± SEM, statistical significance was set to *P* < 0.05, and all nonsignificant terms are omitted for clarity.

## 3. Results

### 3.1. WIN-2 Increased NREM Sleep and Reduced Wakefulness: No Reversal by AM251

Our specific interest was in the effects of the full CB1 agonist on sleep and its possible mechanism. Consequently, WIN-2 (1 mg/kg) and AM251 (2 mg/kg) were administered during the sleep phase of the animal (light cycle) and recordings commenced until the start of the nocturnal activity phase. 

**Figure 1:**
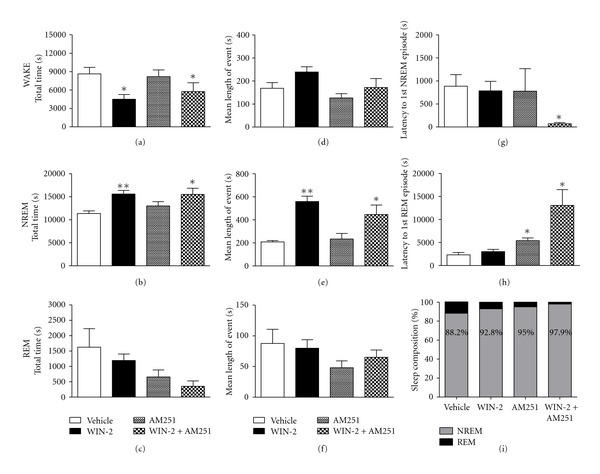
Total time and average length of wakefulness (a, d); NREM (b, e) and REM (c, f) episodes following systemic injections (i.p.) of vehicle (control), WIN-2 (1 mg/kg), AM251 (2 mg/kg), WIN-2 (1 mg/kg)  +  AM251 (2 mg/kg). WIN-2 significantly increased the time spent in NREM sleep whilst decreasing wakefulness; this was not reversed by AM251. Coadministration of WIN-2 and AM251 reduced the latency to the 1st NREM event (g) whilst AM251 either alone or in combination with WIN-2 increased the latency to the 1st REM (h) episode in comparison to controls. The overall sleep composition (% of NREM versus REM) following each respective treatment is depicted in (i). All data (mean ± SEM) were pooled over a 6 h recording period in the light phase. *P < 0.05; **P < 0.01 for paired comparison relative to vehicle treatment.

Sleep was affected by both drugs in different ways. The total time spent awake did not change over all groups (Figure 1(a)), but WIN-2 reduced wakefulness (Figure 1(a)) by about 45%, and this was not reversed by AM251. In contrast, NREM sleep differed reliably between treatments (*F*(3,15) = 4.83, *P* = 0.029) mainly due to heightened NREM amounts in the WIN-2 groups (Figure 1(b)). Again, AM251 alone did not affect this parameter, and there was no reversal of the WIN-2-induced increase in NREM sleep. No significant differences were obtained for REM sleep (Figure 1(c)) despite some reductions after WIN-2 + AM251 injections. Although there was some variability between treatment conditions, the length of each wakefulness or REM sleep episode was not affected by any of the drugs (Figures 1(d) and 1(f)). However, the average length of NREM bouts differed reliably between treatments (*F*(3,15) = 11.84, *P* = 0.002; Figure 1(e)) and was considerably increased in both WIN-2 groups. AM251 again had no effect.

Interestingly, the latency to first sleep (NREM and REM) episodes after drug treatment was not affected by WIN-2, but AM251 + WIN-2 treatment prolonged the latency to 1st REM (*t* = 3.11, *P* < 0.05; Figure 1(h)) whilst decreasing the latency to 1st NREM episode (strong trend: *P* > 0.05 < 0.1; Figure 1(g)). A main effect of treatment (*F*(3,15) = 6.77, *P* < 0.05) was found for latency to 1st REM episode only.

Finally, both WIN-2 and AM251 affected the overall sleep composition (Figure 1(i)). Under vehicle control conditions, animals were in NREM sleep approximately 88.2% of total sleep time (i.e., 11.8% REM sleep). However, treatment with WIN-2, AM251, and WIN-2 + AM251 increased the total NREM sleep time to 92.8%, 95%, and 97.9%, respectively. These data suggest additivity of suppressing REM sleep for WIN-2 and AM251 and imply differential underlying mechanisms.

### 3.2. WIN-2 Reduced While WIN-2 + AM251 Enhanced Spectral Power Globally

**Figure 2:**
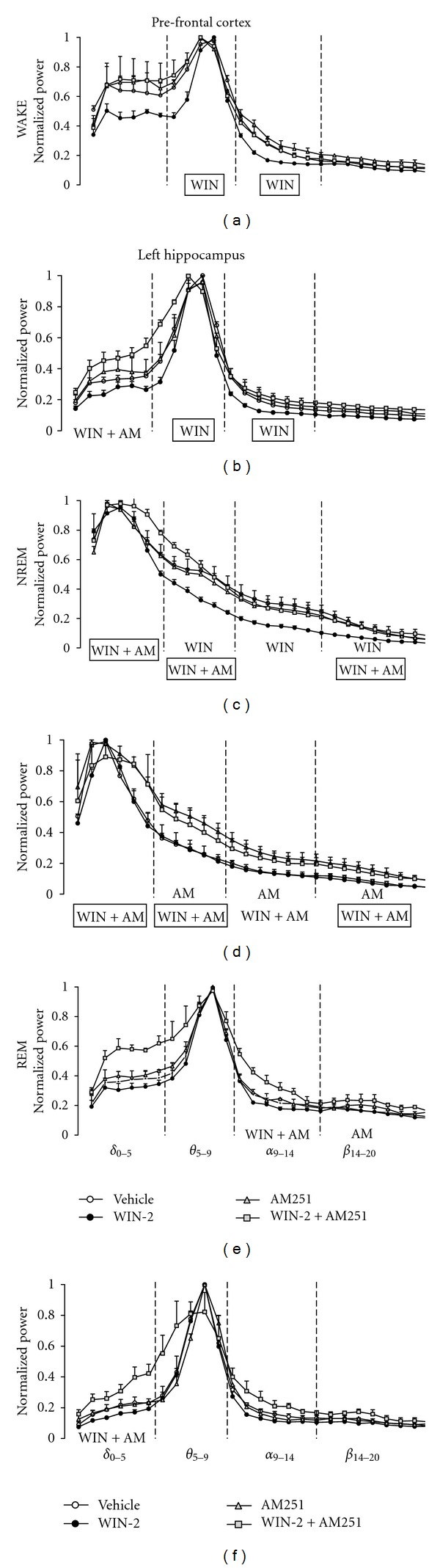
Normalized EEG power spectra recorded by electrodes positioned above the prefrontal cortex and parietal cortex/dorsal (left) hippocampus (frequency bands as indicated in e and f), following systemic treatment with vehicle (control), WIN-2 (1 mg/kg), AM251 (2 mg/kg), or WIN-2 (1 mg/kg) + AM251 (2 mg/kg) for vigilance stages of wakefulness (a and b), NREM (c and d) and REM (e and f) sleep, respectively. Significant effects of treatment are depicted as WIN (WIN-2), AM (AM251), and WIN + AM (WIN-2 + AM251) for respective frequency bands in each subfigure (a–f). Rectangular markings indicate effects common to both prefrontal and parietal recording sites. The normalized power for all data points (0.77 Hz increments from 0 to 20 Hz) is represented as mean ± SEM.

In all vigilance stages and recording sites, WIN-2 produced a reduction in normalized power across all frequency bands (Figure 2; only left parietal recording shown). This was particularly marked during wakefulness and NREM sleep, and less obvious during REM sleep. By contrast, AM251 alone enhanced power during NREM, but had little or no effect during wakefulness and REM sleep. When both compounds were coadministered, spectral power was greatly enhanced in NREM and REM sleep in both prefrontal and parietal recording sites. These observations were confirmed by statistical analysis. Treatment-related differences (and drug-by-frequency interactions) were significant for all areas and vigilance stages (all *F*'s >2; *P*'s <0.05) justifying a more in-depth post hoc analysis of planned comparisons between vehicle and drug treatment conditions.

WIN-2 suppressed both theta and alpha power in prefrontal and parietal recordings during wakefulness (all *F*'s >7, *P*'s <0.05; Figures 2(a) and 2(b)), theta, alpha, and beta power during NREM sleep prefrontally (*F*'s >5.5, *P*'s <0.05; Figure 2(c)), but not at parietal sites (Figure 2(d)), and had no effect on REM spectral power. By contrast, AM251 alone had highly vigilance stage and region-specific effects. It did not affect spectral power during wakefulness or REM sleep, but enhanced theta, alpha, and beta power during NREM in parietal (*F*'s >6; *P*'s <0.05; Figure 2(d)), but not in prefrontal cortex.

Based on behavioural results [15, 17, 20], we expected AM251 to reverse some of the effects of WIN-2. Indeed, WIN-2-induced reductions in spectral power during wakefulness in prefrontal cortex were prevented by coadministration of AM251 (no reliable difference to vehicle; Figure 2(a)) or converted into an enhancement in delta power for parietal recording sites (*F*(1,30) = 6.38, *P* < 0.05, Figure 2(b)). A similar heightening of delta power was also found for NREM and REM (together with alpha power increase) episodes when both drugs were injected conjunctively (*F*'s >6.4; *P*'s <0.05; Figures 2(c), 2(d), 2(e), and 2(f)). Finally, WIN-2 and AM251 together increased power in parietal cortex during NREM also in theta, alpha, and beta bands (*F*'s >2.5; *P*'s <0.05;Figure 2(d)) to levels similar to AM251 alone.

These effects clearly differ from alterations observed for each drug alone and confirm that AM251 indeed is able to prevent WIN-2-induced suppressions in power in both prefrontal and parietal cortex, suggesting that WIN-2-dependent alterations in theta/alpha power during wakefulness are likely to be mediated by CB1 receptor activation (see Figures 2(a) and 2(b)). By contrast, the AM251-related increase in spectral power in theta, alpha, and beta bands during NREM was not reversed in the presence of WIN-2 in the hippocampus, which suggests that these changes were not mediated by CB1 receptors.

## 4. Discussion

This study is the first effort to characterize the pharmacological effects of the full CB1 receptor agonist, WIN-2, and antagonist/inverse agonist, AM251, either alone or in combination on sleep patterns in rats. The acute effects of these cannabinoids on sleep were assessed using epidural EEG recordings through wireless microchip technology, which has previously only been applied to mice [18, 21]. This novel technology produced results similar to previous work on other CB1 receptor agonists using tethered EEG recordings [1]; we report alterations in sleep-wake architecture by enhancing NREM sleep mainly at the expense of wakefulness following cannabinoid administration.

WIN-2-induced disruptions in normal sleep profiles in rats are consistent with other studies that have shown considerable increases in NREM (sometimes also referred to as slow wave sleep SWS) sleep with reduced wakefulness following the administration of anandamide and Δ^9^-THC [1, 12–14, 22]. These studies also indicated CB1 receptor activation as the underlying mechanism [13, 14, 22]. Our own data from coadministration of WIN-2 with AM251 in rats suggest, however, that there are CB1-dependent and CB1-independent actions exerted by the full agonist. The overall reduction in spectral power in theta, alpha, and beta bands was reversed by AM251 providing evidence for CB1-receptor modulation of global spectral power. At the same time, AM251 failed to reverse the WIN-2-induced enhancement in NREM sleep alongside the decrease in wakefulness implicating non-CB1 receptor-mediated mechanisms in the modulation of sleep. It is conceivable that WIN-2 also activated other cannabinoid sensitive targets such as CB2 or non-CB1/CB2 receptors [23–26], but their influence on vigilance stages remains to be determined.

Intriguingly, AM251 alone failed to produce major alterations in vigilance stages in comparison to controls, except for a subtle prolongation of the latency to 1st REM episode and enhanced spectral power at parietal recording sites specifically during NREM sleep. A greater prolongation of the latency to 1st REM episode and heightened power in lower frequency bands was apparent when AM251 was coadministered with WIN-2. These effects, however, cannot be ascribed to any receptor actions at present and may even comprise a mixture of effects on different effector systems. Important to note is the fact that AM251 behaved differently to SR. While the former was without influence on vigilance parameters, SR not only delayed REM sleep onset but also increased wakefulness and reduced NREM and REM sleep [8, 14]. This discrepancy may be a result of different pharmacological profiles between these two compounds [27]. A direct comparison with human marijuana users is difficult given the repeated exposure to cannabis in most previously published studies. Acute Δ^9^-THC, however, increased total slow wave sleep in humans [28] and increased latency to sleep onset when given in conjunction with equal concentrations of cannabidiol [29], a phytocannabinoid readily bioavailable to the brain [30] and antagonizing some of the effects of CB1 agonists in vivo [31]. In keeping with these observations, coadministered WIN-2 and AM251 also prolonged the onset of REM sleep specifically confirming the overall disruption of the normal sleep signature after cannabinoid exposure.

A reduction in normalized power following WIN-2 treatment during wakefulness suggests a lowering in neuronal synchrony in hippocampal-cortical projections. At the same time, the selective loss of power in theta (5–9 Hz) and alpha (9–14 Hz) frequency bands during wakefulness may explain why rats' performance in working/short-term memory paradigms [15, 20, 32, 33] is compromised, and hippocampal cell ensemble firing during task-specific events (i.e., encoding) is lost in the presence of WIN-2. While WIN-2 increased the overall amount and bout length of NREM episodes, which is presumed to support consolidation of episodic-like memory [34], this clearly cannot compensate for any deficit in encoding. Higher amounts of NREM also led to a disruption of sleep architecture. Since these alterations were not sensitive to AM251 cotreatment, they are unlikely mediated through direct actions on CB1 receptors. Although speculative at this stage, indirect modulation of the cholinergic system by cannabinoids such as WIN-2 should be considered in future studies, because cholinergic activity plays a key role in regulating sleep [35] and its stimulation can prevent memory deficits induced by WIN-2 [15, 17]. However, alternative pathways such as activation of phospholipase C [13, 36] or production of adenosine [22, 36] cannot be ruled out at this stage.

Collectively, our data support the sleep promoting ability of WIN-2 and may also explain some of the working/short-term memory deficits seen after administration of this drug [15, 33] as they have been related to hippocampal prefrontal interactions during active performance in learning tasks. Although a lowering in normalized spectral power appeared to be mediated by CB1 receptors, WIN-2 effects on sleep in rats appear to be non-CB1-mediated suggesting alternative drug effects [15, 17] and warranting future in-depth investigations to determine mechanisms of actions of the different cannabinoids.

##  Conflict of Interests

The authors report no conflict of interests.
